# Superconfinement tailors fluid flow at microscales

**DOI:** 10.1038/ncomms8297

**Published:** 2015-06-15

**Authors:** Siti Aminah Setu, Roel P.A. Dullens, Aurora Hernández-Machado, Ignacio Pagonabarraga, Dirk G.A.L. Aarts, Rodrigo Ledesma-Aguilar

**Affiliations:** 1 Department of Chemistry, Physical and Theoretical Chemistry Laboratory, University of Oxford, South Parks Road, Oxford OX1 3QZ, UK; 2 Department of Chemistry, Faculty of Science, Universiti Teknologi Malaysia, Johor Bahru, Johor 81310, Malaysia; 3 Departament d'Estructura i Constituents de la Matèria, Universitat de Barcelona, C. Martí i Franquès 1, Barcelona E-08028, Spain; 4 Departament de Física Fonamental, Universitat de Barcelona, C. Martí i Franquès 1, Barcelona E-08028, Spain; 5 The Rudolf Peierls Centre for Theoretical Physics, University of Oxford, 1 Keble Road, Oxford OX1 3NP, UK; 6 Oxford Centre for Collaborative Applied Mathematics, Mathematical Institute, University of Oxford, Radcliffe Observatory Quarter, Woodstock Road, Oxford OX2 6GG, UK; 7 Department of Physics and Electrical Engineering, Northumbria University, Ellison Place, Newcastle upon Tyne NE1 8ST, UK

## Abstract

Understanding fluid dynamics under extreme confinement, where device and intrinsic fluid length scales become comparable, is essential to successfully develop the coming generations of fluidic devices. Here we report measurements of advancing fluid fronts in such a regime, which we dub superconfinement. We find that the strong coupling between contact-line friction and geometric confinement gives rise to a new stability regime where the maximum speed for a stable moving front exhibits a distinctive response to changes in the bounding geometry. Unstable fronts develop into drop-emitting jets controlled by thermal fluctuations. Numerical simulations reveal that the dynamics in superconfined systems is dominated by interfacial forces. Henceforth, we present a theory that quantifies our experiments in terms of the relevant interfacial length scale, which in our system is the intrinsic contact-line slip length. Our findings show that length-scale overlap can be used as a new fluid-control mechanism in strongly confined systems.

## Abstract (web summary)

An understanding of how fluid–solid interactions affect flow dynamics is essential for microfluidics design. Here the authors show how fluids in linear channels can be controlled by the degree of confinement when the contact-line slip length is comparable to the channel size.

## Graphical abstract

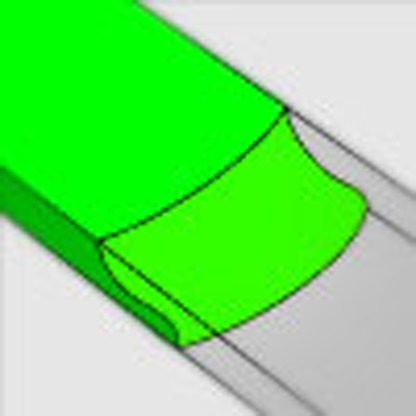

The ability to control the motion of fluids in nano- and microfluidic devices is of fundamental and practical interest in a variety of fields^1^,^2^ with promising applications that range from health^3^ to materials science^4^. Confinement plays a primary role in microfluidic control: hydrodynamic interactions between free and stationary solid boundaries can be used, for example, to steer suspended colloids according to their shape^5^. On the other hand, the competition between viscous and capillary effects mediated by channel geometry can be used to manipulate interfacial structures. Drops are arguably the most exploited fluid structures in microfluidics, acting, for example, as carriers and mixers^6^. Precise production and manipulation of drops demands a quantitative understanding of how solid–fluid interactions affect flow patterns, including the effect of the channel walls and wetting^7^,^8^.

Current drop-based microfluidic devices rely on nonlinear channel geometries, such as T-junctions and flow focusing constrictions, to induce the pinch-off of droplets from a mother stream (Fig. 1a). A common trait of these set-ups is that they operate in the well-developed hydrodynamic limit. In such a case, the length scale of the confining devices is well above any microscopic length scale of the fluid. As a consequence, one expects that the dominant contributions to the fluid dynamics come from forces acting on the volume of the fluid, such as pressure gradients and viscous friction forces. The limit of sharp length-scale separation breaks down as one enters the nanofluidic regime^9^, or, equivalently, in microfluidic systems containing complex fluids, such as colloidal mixtures, liquid crystals and bacterial suspensions, where the ‘molecular' size can be of the order of microns. For such strongly confined systems one expects that the dynamics is increasingly dominated by the interaction with confining walls. Specifically, for fluid–fluid–solid systems the range of interfacial forces can be characterized in terms of a contact-line slip length, *l*_D_, which is an intrinsic length scale of the solid–fluid system^10^,^11^,^12^. Contact-line slip affects the stability of moving fronts, as it determines the critical speed at which the contact line can move before it lags behind the rest of the fluid front^13^. Such an entrainment mechanism is determined by the small-scale motion of fluid molecules flowing past the solid near the contact line, which determines how fast it can move^14^. For large systems, the separation between *l*_D_ and the typical length scale of the flow has been shown to affect the way in which solid–fluid interactions control the critical speed^15^,^16^. This has helped to explain complex interface dynamics reported in a variety of experiments^16^,^17^,^18^,^19^,^20^. However, little is known about how multiphase systems respond under extreme confinement, where the system size and the contact-line slip length become comparable.

In this paper we exploit the dominant role of interfacial forces in superconfinement to control fluid flow at small scales. As shown in Fig. 1b, by forcing a phase-separated colloid–polymer mixture on the bottom wall of a microchannel, the three-phase contact line can be slowed down relative to the leading front, destabilizing the interface. The critical speed at which such a transition occurs, U*, decreases with increasing channel thickness *H* (data points in Fig. 1b). This allows us to control the formation of liquid jets and drops by varying the degree of confinement in linear microchannels. Our concept thus departs from a paradigm in microfluidics, which is the use of complex geometries to achieve fluid control. Using numerical simulations we show that the fluid dynamics is controlled by the interplay between the intrinsic contact line slip length and the thickness of the microchannel. This allows us to formulate a scaling model that predicts a strong decay of the critical velocity with the channel thickness, in agreement with the experimental data (solid line in Fig. 1b). Our experiments show that by controlling the degree of confinement, it is possible to trigger the formation of different fluid structures, including drops, jets and drop-emitting fingers.

## Results

### Front instability induced by superconfinement

Our experimental results are summarized in Fig. 2, where we have probed the dynamics of a superconfined forced front coupled to a contact line using two demixed colloid–polymer phases. The interface thickness of our mixtures, *ξ*, which is representative of the order of magnitude of the contact-line slip length *l*_D_, is of the order of microns (*ξ*≈1.2 μm). This allows us to access the superconfined regime in micron-sized channels. As opposed to more traditional set-ups, the large interface scale in our experiments offers the additional advantage of directly visualizing the front and the contact line at the scale of thermal fluctuations using confocal microscopy^21^,^22^. To favour the formation of a fluid front advancing on a solid, we injected two co-existing colloid–polymer phases into a rectangular microfluidic channel, displacing the more viscous polymer-rich, colloid-poor ‘gas' phase with the less viscous polymer-poor colloid-rich ‘liquid' phase (Fig. 2a). The low surface tension of our mixtures, *γ*=30 nN m^−1^, sets a comparatively small capillary length, 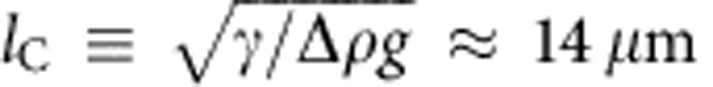, where Δ*ρ* is the density difference between the fluids and *g* is the acceleration due to gravity. At large enough velocities, the interface develops into a three-dimensional (3D) finger by virtue of the Saffman–Taylor instability^22^ (Fig. 2b). Because *l*_C_ is of the order of the channel thickness *H*, the finger adheres to the bottom plate^22^ creating a thin film of thickness *h*_f_, which occupies roughly half of the channel thickness (Fig. 2c).

**Figure 1:**
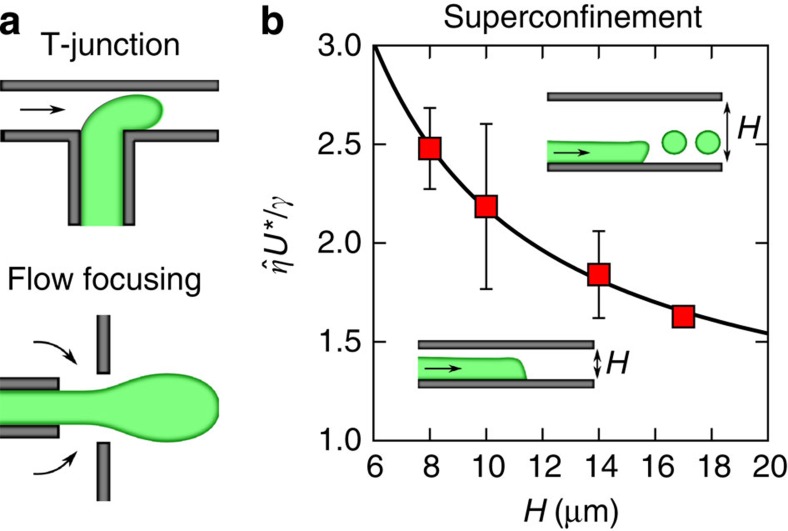
Drop production in standard and superconfined microfluidic set-ups. (**a**) Traditional microfluidic set-ups rely on drop geometry to trigger the formation of drops. (**b**) In superconfinement, the ability of a forced liquid front to cover a microchannel wall can be controlled by varying the thickness of the channel. Our experimental results (symbols) show that drops are produced above a critical driving velocity U*, which can be controlled by varying the thickness of the microchannel, *H*. The solid line corresponds to the theoretical prediction (see text). Speeds are measured in units of the capillary speed, 
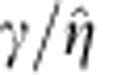
, where *γ* is the interfacial tension and 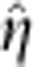 is the mean viscosity of the fluids (see Methods for further information on the experimental set-up and data analysis).

**Figure 2:**
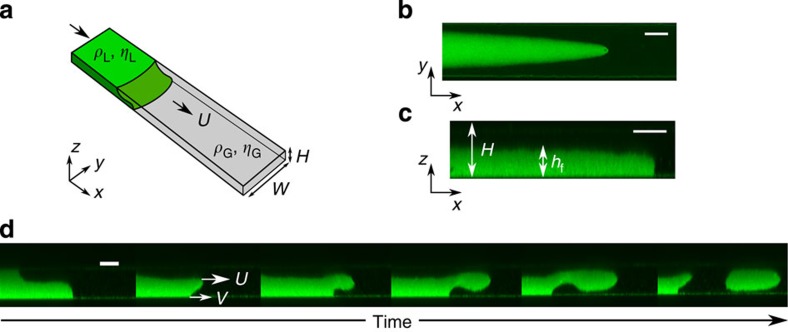
Interface dynamics in superconfinement. (**a**) Two colloid–polymer phases of ultra-low surface tension, *γ*=30 nN m^−1^, and different densities (*ρ*_L_>*ρ*_G_) and viscosities (*η*_L_<*η*_G_), are forced in a microfluidic channel of fixed width *W*=110 μm and variable thickness *H*=8, 10, 14, 17 μm. (**b**,**c**) The interface in the plane of the channel develops into a viscous finger (**b**), which adheres to the bottom plate of the channel to form a thin film (**c**). (**d**) Above a threshold driving speed, U*, the front is destabilized; the contact line, of speed *V*<U*, is unable to follow the rest of the interface, of speed *U*>U*. This mismatch gives rise to the formation of a fluid jet that releases drops periodically. Scale bars in **b**–**d**, 50, 10 and 10 μm, respectively.

The interface reaches a well-defined maximum speed U*, at which point entrainment occurs (Fig. 2d and Supplementary Movie 1). Henceforth, the contact line, of speed *V*, moves slower than the leading front, of speed *U* (Fig. 2d; left panels). This mismatch causes the interface to develop into a jet whose neck collapses, giving rise to the periodic release of droplets (Fig. 2d; right panels). The critical driving speed is strongly dependent on confinement and decays with the channel thickness (Fig. 1b).

### Hydrodynamics of superconfined fronts

To gain insight into the mechanisms governing the front dynamics at length scales comparable to the interface scale, we carried out numerical simulations of the mesoscopic hydrodynamic equations of a binary fluid model. The model is valid to describe the fluid dynamics at length scales larger than, but still comparable to, the interface thickness *ξ* (refs 10, 11, 12). In our simulations, the composition of the colloid–polymer mixture is described by a single concentration variable, *φ*, which varies between two equilibrium values over the length scale of the interface, *ξ*. As usual, the fluid dynamics is governed by mass and momentum conservation. For incompressible flows, this leads to the following system of equations:


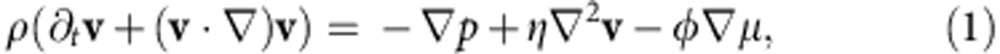


and


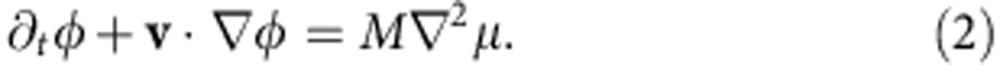


Equation (1) is the usual Navier–Stokes equations for an incompressible fluid, where *ρ* is the fluid density, *η* is the viscosity and *p* and **v** are the pressure and velocity fields, respectively. The last term on the right-hand side includes the interfacial contributions to the dynamics. It depends on the chemical potential *μ* and gives rise to interfacial and contact line forces. The dependence of the chemical potential on the concentration field is determined by the equilibrium properties of the system. In the present case, we resort to the well-known Ginzburg–Landau model, for which 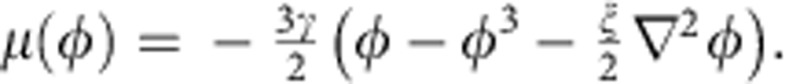 Equation (2), known as the Cahn–Hilliard convection-diffusion equation, governs the transport of the concentration field *φ*. Advective transport arises from the coupling term on the left-hand side. Diffusive transport arises from inhomogeneities in the chemical potential field, represented by the term on the right-hand side, and is controlled by the mobility parameter *M*.

**Figure 3:**
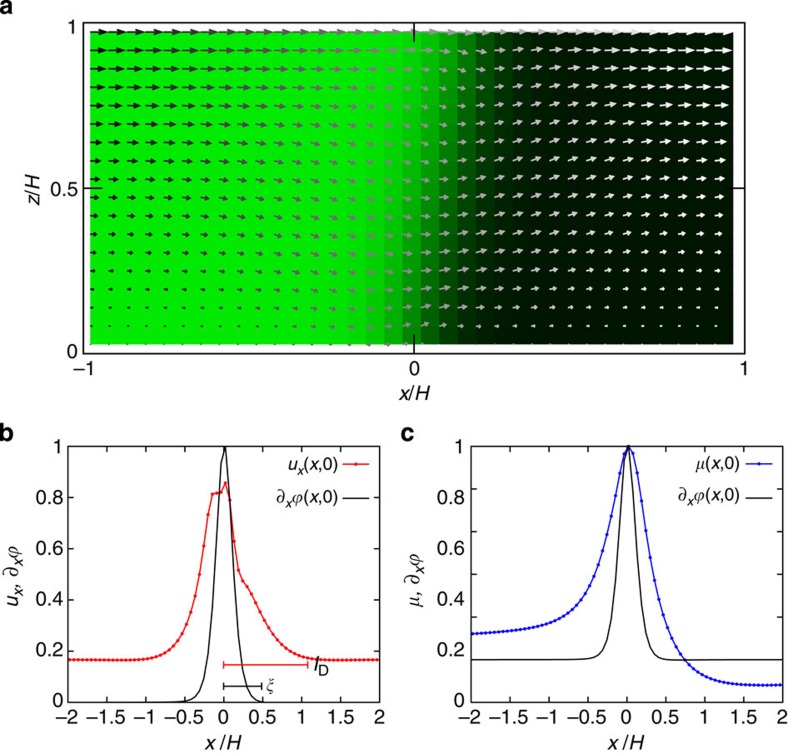
Numerical simulations of flow patterns in superconfinement. (**a**) The concentration and velocity fields for a forced interface between parallel plates. The concentration profile of two demixed phases (colour intensity map) varies across the interface length scale *ξ*. The colloid-rich phase is represented in bright green and the polymer-rich phase in dark grey. The arrows represent the velocity field, which is homogeneous over length scales comparable to the system size, including the region in contact with the stationary bottom wall. (**b**) Velocity profile and concentration gradient close to the wall. The slip velocity deviates from the stick boundary condition over a length scale, *l*_D_, larger than the interface length scale *ξ*. (**c**) Chemical potential profile along the wall. The slip profile originates from deviations of the chemical potential, *μ*, from equilibrium. The length scales over which deviations decay is given by the same length scale *l*_D_.

**Figure 4:**
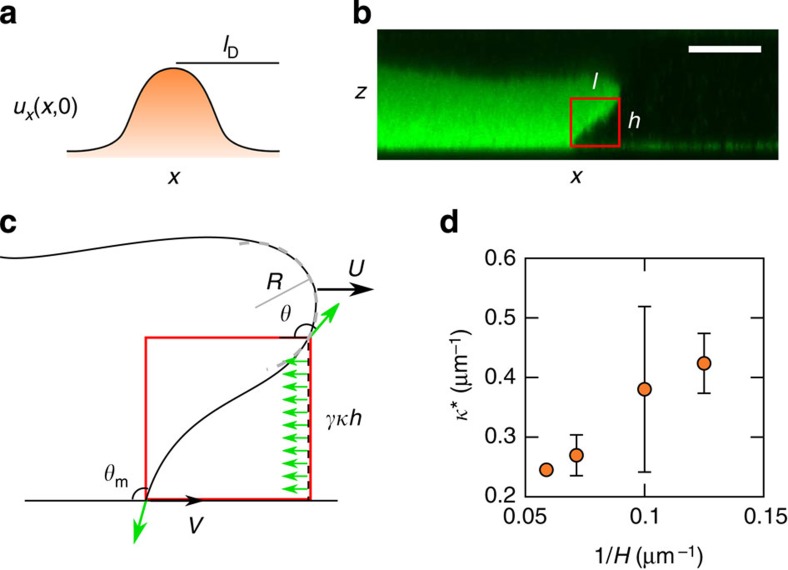
Contact-line speed and interface configuration at the onset of entrainment. (**a**) Schematic of the slip velocity profile, *u*(*x*, *z*=0), close to the contact line. The fluid velocity decays at distances comparable to the contact-line slip length, *l*_D_. (**b**) Interface configuration at the critical speed in a 14-μm-thick channel, just before drop emission. (**c**) Schematic representation of the interface configuration close to the contact line (amplified from frame **b**). The shape of the interface is characterized by the local curvature *κ*=1/*R*, where the radius of curvature is measured at the tip of the front, the bending angle *θ* and the microscopic contact angle *θ*_m_. The surface tension and Laplace pressure forces arising from the deformation of the interface are shown as light arrows. The contact line and front speeds, *V* and *U*, are indicated as dark arrows. (**d**) Critical curvature of the interface as a function of the channel thickness. The curvature was measured from the experimental interface profiles by fitting a circular arc at the tip of the front. Scale bar in **b**, 10 μm. Error bars correspond to the s.d. of the sample.

Using a lattice-Boltzmann numerical algorithm (see Methods), we integrated equations (1) and (2) for a front forced between two parallel plates (see Fig. 3). We resort to two-dimensional (2D) simulations that capture the relevant contact-line physics^12^,^19^. The concentration and velocity fields are shown in Fig. 3a. The scale of the interface is clearly visible; to mimic the experimental conditions we fixed its value to be comparable to the separation between the plates, *H*. In such a regime, the simulations show that the velocity field close to the interface is homogeneous and does not vary appreciably over length scales comparable to the channel thickness. The slip velocity, which corresponds to the tangential component of the velocity field to the wall, deviates from the stick boundary condition over length scales comparable to the channel thickness (Figs 3b and 4a). This sharply contrasts with macroscopic systems, where such deviations occur over length scales much smaller than the channel thickness and are thus not expected to contribute dominantly to the overall fluid dynamics. In the superconfined regime this picture changes. Figure 3b shows the velocity profile close to the wall. The velocity peaks at the contact line and decays over a length scale *l*_D_, which is larger than the interface thickness (indicated in the figure by the width of the gradient of the concentration field). From the simulations, the ratio of the two length scales was found to be *ξ*/*l*_D_≈0.42. The local deviation from the stick boundary condition arises from the imbalance in the chemical potential caused by the deformation of the interface, which allows the contact line to move by virtue of diffusive transport^11^,^12^. The chemical potential deviations from equilibrium decay over the same length scale *l*_D_ as shown in Fig. 3c.

### Stability of superconfined fronts

On the basis of the numerical results, we are in a position to formulate a scaling model to predict the critical velocity in superconfinement. In our experiments, the front is driven by a fixed pressure gradient that results in a strong deformation of the interface (Fig. 4b). Close to the solid, this deformation offsets the contact angle from its equilibrium value *θ*_e_ inducing a Young's force, *F*_Y_=*γ*(cos*θ*_e_–cos*θ*_m_), that pushes the contact line. Note that *F*_Y_ depends on deviations of the interface from its equilibrium configuration (*θ*_m_=*θ*_e_) through the microscopic contact angle *θ*_m_ (Fig. 4c). The driving force is opposed by the contact-line friction force, *F*_CL_∼*ζV*, which arises from the sliding of fluid molecules past the solid and thus depends on their speed *V*. The friction coefficient 
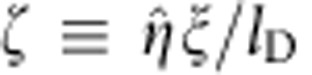
 characterizes the extent over which slip occurs, and is fixed by *ξ*, *l*_D_ and the average viscosity 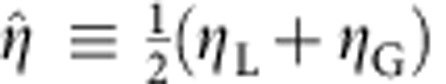.

The contact-line speed *V* is set by the local balance (per unit length of the contact line) between *F*_Y_ and *F*_CL_, leading to the contact-line friction law^13^,^15^,^23^


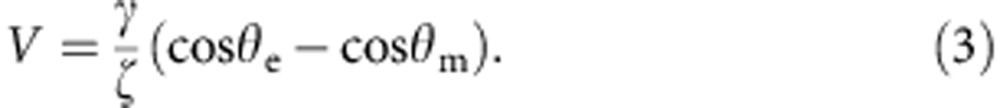


The speed of the front, on the other hand, is set by a global balance (also per unit length of the contact line) between the viscous and capillary forces acting on the fluid wedges that meet at the wall (boxed region in Fig. 4b). At scales comparable to *l*_D_, the fluids are pushed by the capillary force density, ∼∂_*x*_*γκ*, arising from spatial variations of the local interface curvature *κ*. This force is resisted by the viscous force density coming from both phases, ∼*η*_*i*_∇^2^*u*, where *i*={L, G}. From our numerical results, we expect 
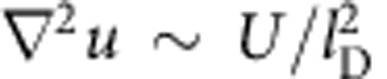
. Integrating the force densities over the size of the liquid and gas wedges then gives


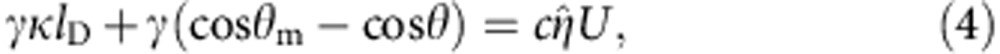


where *θ* is the local bending angle of the interface. The first two terms in equation (4) correspond to the net capillary force, which consists of the Laplace pressure exerted by the fluid lying in front of the wedge, and of two tension forces pulling on the corners (light arrows in Fig. 4c). The right-hand side corresponds to the total viscous force originating from the flow within each fluid. Because the viscosities of both fluids are of the same order of magnitude, the total viscous force, corresponding to the right-hand side of equation (4) can be modelled as a single term, where *c* is a numerical prefactor.

At the onset of entrainment the contact-line speed reaches a maximum, *V*=*U*=U*. In contrast with unbounded fronts, where the profile can adjust freely to the external driving, in superconfinement we expect that the critical interface curvature is set by the channel thickness, that is, κ*∼1/*H*. To confirm this argument, we measured the critical interface curvature at the tip of the front. Results are shown in Fig. 4d, which confirms the linear relation κ*=*aH*^−1^, with *a*≈3.6. Eliminating *θ*_m_ from equations (3) and (4) we find that the critical speed obeys


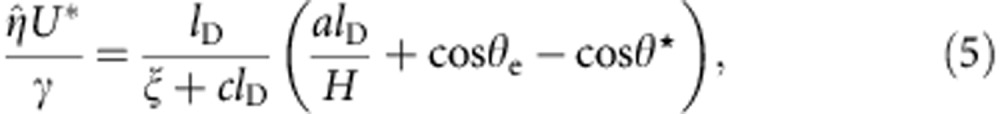


where θ* is the critical bending angle.

Equation (5) predicts an algebraic decay of the critical speed with the channel thickness, consistent with our experiments (Fig. 1b). The algebraic dependence on the system size, *H*, arises from the linear scaling of the critical radius of curvature with the channel thickness, which sets the maximum deformation that the interface can sustain before destabilizing. This contrasts with previous predictions of entrainment in open^15^ and confined systems^24^ at large length-scale separations (*H*≫*l*_D_) where the dependence of the critical velocity with the system size is controlled by volume contributions to viscous friction, scaling as ln(*H*/*l*_D_). In superconfinement, where *H*≃*l*_D_, such contributions are subdominant. Instead, the relevant contribution to friction is dominated by the contact line, which is independent of the system size.

To compare the theoretical prediction with the experiments we define the threshold speed at the point where *θ* reaches 90°, which is the point where the front begins to destabilize. This criterion can underestimate the value of the true critical angle θ*, but does not affect the scaling with the confining length scale *H*, which is the matter of interest. A best fit of the data shown in Fig. 1b to equation (5) allows us to extract the contact-line slip length and the prefactor *c* as fitting parameters. We find *l*_D_≈3.8 μm. The ratio *ξ*/*l*_D_≈0.32 is in good agreement with the value measured from our numerical simulations. Recently, it has been shown that small values of *ξ*/*l*_D_ (compared with unity) can strongly reduce the large-scale stability of forced fronts by fixing the shape of the interface close to the contact line^15^. Such an interplay between contact-line and bulk friction can give rise to different entrainment regimes depending on the wetting properties of a given solid–fluid system. Using superconfinement to estimate the contact-line friction coefficient can therefore prove useful to exploit surface specificity in controlling front dynamics.

### Tailoring structures, jet formation and drop emission

**Figure 5:**
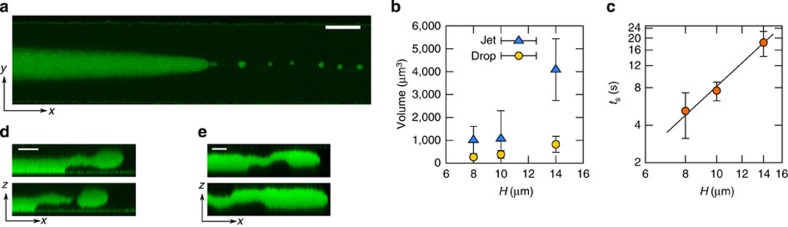
Jet formation and drop emission in superconfinement. (**a**) Periodic emission of drops above the onset of entrainment. (**b**) Volume of the jet, and of the emitted drops, as a function of the channel thickness. The larger jet volume value at *H*=14 μm reflects the long snap-off time of the neck at large *H* relative to the interface thickness. (**c**) The snap-off time increases algebraically with *H*. (**d**) Growth of a liquid jet from the contact line (top) and collapse to release a drop (bottom) at *H*=14 μm. (**e**) Stabilization of the emitted jet at *H*=17 μm. Scale bars in **a**,**d**,**e**, 50, 10 and 10 μm, respectively. Error bars correspond to the s.d. of the sample.

Beyond the onset of entrainment, the interface also responds to changes in confinement. This is reflected in the periodic emission of drops shown in Fig. 5a and Supplementary Movie 2, whose size and rate of release can be controlled by choosing the channel thickness (Fig. 5b,c). Drop emission begins with the slow down of the contact line relative to the front, which triggers the growth of a liquid jet. For small *H*, when the jet and interface thicknesses are comparable, the jet develops a thinning neck that connects to a nascent drop, which is then released (Fig. 5d). Because the speed of the front is kept above the entrainment threshold, subsequent drops are emitted at a constant rate.

The volume of the drops (Fig. 5b) is fixed by the snap-off process, which sets the typical fluid volume flowing into the jet (of thickness ∼*h*_f_) over the snap-off time *t*_s_, that is, 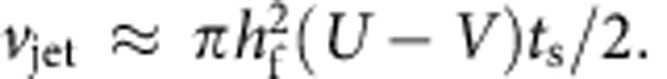 As shown in the figure, *v*_jet_ is generally larger than the volume of the drops, which we estimate assuming an ellipsoidal shape as *v*_drop_≈4*π*(2*r*_||_)^2^(2*r*_∥_)/3 where *r*_||_ and *r*_∥_ are the drop radii of curvature in the parallel and perpendicular planes to the wall. The mismatch between *v*_jet_ and *v*_drop_ is caused by the typically long necks that develop, which are absorbed by the main front once the drop is released.

The growth of the drop size with *H* observed in Fig. 5b arises because both *h*_f_ 
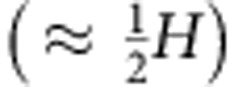
 and *t*_s_ increase with the channel thickness. Since the thickness of the neck connecting the jet to the drop, *h*_n_, is comparable to the interface thickness *ξ*, we expect that its collapse is dominated by thermal fluctuations. This contrasts with drop emission at large scales, where the neck collapse is dominated by hydrodynamics^19^. The fluctuation-dominated neck breakup is characterized by double-cone jet profiles^25^,^26^,^27^ and by a faster (algebraic) decay than that of macroscopic jets, which is linear^28^. Following ref. 29, the collapse of the neck obeys *h*_n_(*t*)∼(*t*_s_–*t*)^*α*^, where *α*≈0.42. Since *h*_n_∼*H*, we expect that the snap-off time obeys *t*_s_∼*H*^1/*α*^. This prediction is tested against the experimental data in Fig. 5c, showing a good agreement. Remarkably, for the thicker channel used in our experiments (*H*=17 μm) the jet is stabilized after one snap-off event and no further drops are emitted in the timescale of the experiment (Fig. 5e). This suggests a cross-over to macroscopic hydrodynamics where viscous stresses coming from the flow within the jet hamper its collapse.

**Figure 6:**
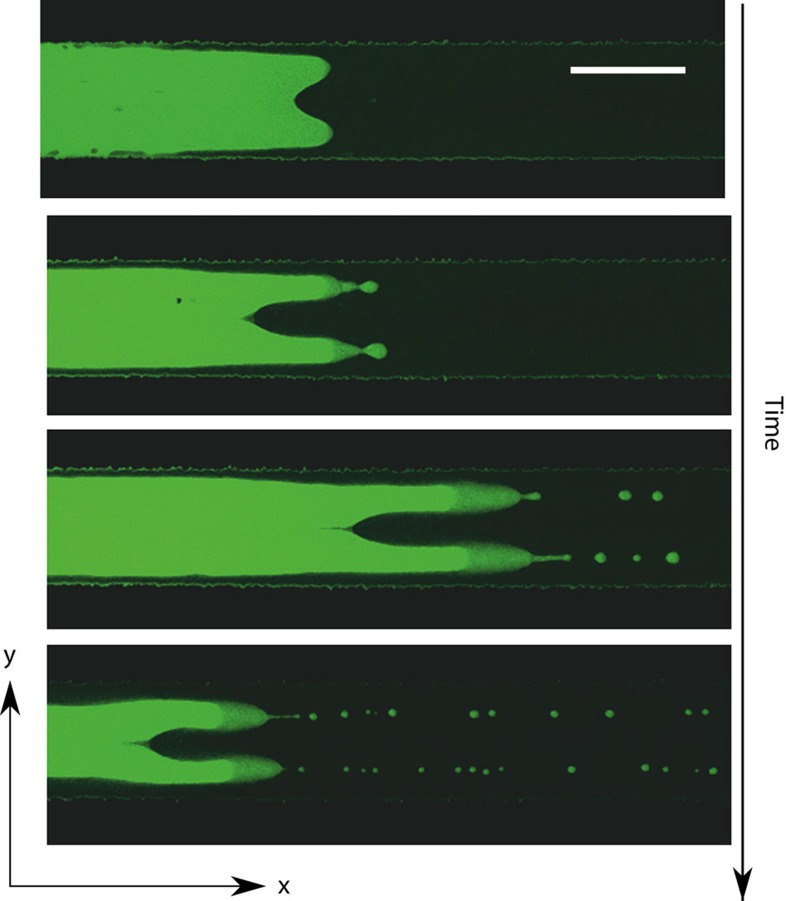
Tip-splitting and concurrent drop emission. At high values of the capillary ratio, 
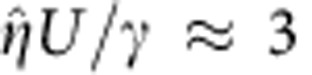
, the front splits into two fingers that subsequently destabilize and emit drops periodically. Scale bar, 100 μm.

The combination of low surface tension and strong confinement in our set-up can be further exploited to induce the formation of complex structures; by quickly forcing the interface into our microchannels, it is possible to trigger the formation of pairs of fingers that subsequently emit drops concurrently (Fig. 6 and Supplementary Movie 3). These experiments indicate the feasibility of manipulating fluids through the combination of low surface tension and superconfinement, and open the possibility of controlling the stability and synchronization of the emerging structures.

## Discussion

We experimentally probe the dynamics of fluid mixtures in superconfinement, allowing us to extract the contact-line slip length and to deduce the contact-line friction coefficient. Because of the overlap between the channel and fluid length scales inherent to superconfinement, our method can be used to infer the microscopic friction processes of fluid mixtures independently from the bulk hydrodynamics. This information can then be used to resolve the coupling between large- and small-scale hydrodynamics in a particular set-up.

Our results show that the interplay between front and contact-line dynamics in superconfinement leads to a distinctive interfacial instability. By choosing the degree of confinement, we have shown that fluid entrainment, jet formation and drop emission can be either induced or suppressed. Such a strategy can be used to control fluid dynamics at small scales.

Because of the universality of length-scale overlap, our findings highlight the need to explore other important phenomena in superconfinement. For instance, our results suggest that dynamic wetting can be controlled by matching the interfacial length scale to the typical feature size of topographically nanopatterned surfaces. Further exploration of hydrodynamic instabilities^30^, superhydrophobicity^31^, elastocapillarity^32^ and active^33^ and soft matter dynamics^34^ in superconfinement can potentially help us harness fluid manipulation at extremely small scales.

## Methods

### Experiments

We used a mixture of fluorescently labelled poly(methyl methacrylate) particles (diameter=210 nm) and non-adsorbing xanthan polymer (molecular weight=4 × 10^6 ^g mol^−1^, radius of gyration=264 nm) dispersed in water^35^. At high enough polymer concentrations, the mixture phase separates spontaneously in a colloid-rich polymer-poor ‘liquid' phase in co-existence with a colloid-poor polymer-rich ‘gas' phase due to the depletion interaction mediated by the polymers^36^,^37^. For the state point used in our experiments the surface tension, *γ*=30 nN m^−1^, and interface thickness, *ξ*≈1.2 μm, were measured from the capillary-wave spectrum^21^. The equilibrium contact angle, *θ*_e_=0°, was determined from the interface profile close to a vertical wall^38^. The viscosity of each phase was measured on a TA AR-G2 rheometer. Both viscosities remained in the Newtonian regime for a wide range of applied shear rates, giving *η*_L_=7 mPa·s for the liquid phase and *η*_G_=15 mPa·s for the gas phase. After the phase separation had completed, we carefully isolated the two phases; the two fluids were subsequently injected into a cross-channel microfluidic device (of fixed width *W*=110 μm and variable thickness *H*=8, 10, 14, 17 μm), at which point they were in co-existence again. The displacing liquid phase was slowly injected to form a flat interface with the resident gas phase at the centre of the cross-channel before a driving pressure gradient was imposed. Gravity was used to control the pressure difference between the inlet and outlet of the channel. The resulting flow was imaged in 3D by means of confocal laser scanning microscopy (Zeiss Exiter), which records the fluorescence of the colloids.

The uncertainty of experimental measurements corresponds to the s.d. of the sample, of size *N*. The experimental data presented in Figs 1, 4 and 5 is presented in Tables 1, 2, 3.

### Numerical

Simulations of equations (1) and (2) were performed using the lattice-Boltzmann algorithm. The cross-section of the microchannel is modelled as a 2D rectangular domain of total area 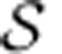. This is discretized into a square lattice composed of nodes joined by links. At each node, we consider two sets of one-particle distribution functions, *f*_*i*_ and *g*_*i*_, each associated with a set of microscopic velocity vectors {*c*_*i*_}. The distribution functions evolve according to the discretized Boltzmann equations


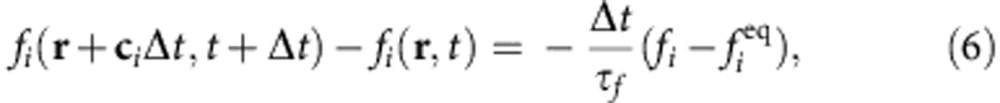


and


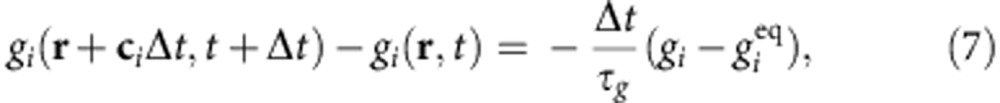


where **r** is the Cartesian position vector and *t* is time. Equations (6) and (7) consist of two steps. First, there is a collision step between fluid particles (right-hand side in the equations), which relax towards an equilibrium state defined by 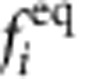 and 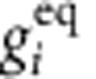. Within the single-relaxation time model used in this paper the equilibration of the *f*_*i*_ and *g*_*i*_ occurs over timescales *τ*_*f*_ and *τ*_*g*_, which in turn determine the viscosity and mobility parameters. Once the collision step has taken place, the distribution functions are propagated along links joining lattice nodes to their first eight nearest neighbours. These are defined by the set of vectors {**c**_*i*_Δ*t*}, where Δ*t*=1 is the time step.

The hydrodynamic variables are defined as 
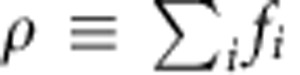
, 
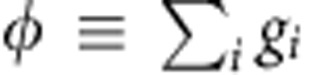
, 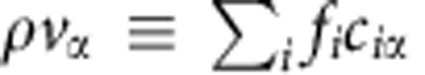 and 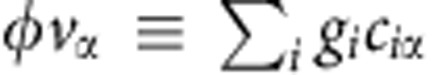, where the Greek index indicates a Cartesian component. Mass and momentum conservation is enforced by imposing the conditions 
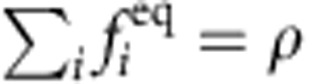
, 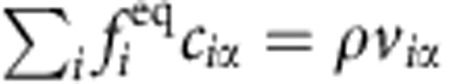, 
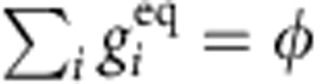
 and 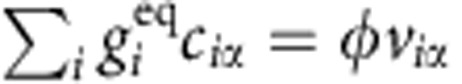. In equilibrium, the pressure tensor and chemical potential are defined through 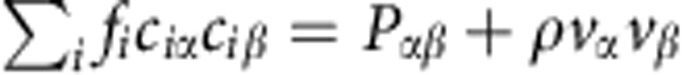 and 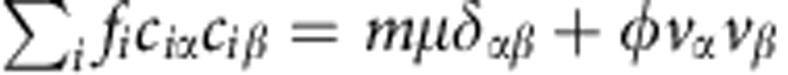, where *m* is related to the mobility parameter in equation (2) and *δ*_*αβ*_ is the Kronecker symbol. The pressure tensor *P*_*αβ*_ contains the isotropic contribution of the bulk pressure and the interfacial stresses.

The equilibrium state of the system is based on the free-energy functional


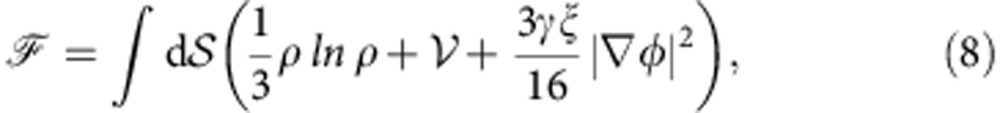


where the first two terms correspond to the the volume contributions to the free-energy density and the last term is the interfacial free-energy density. The colloid–polymer mixture is modelled by way of the Ginzburg–Landau free energy, 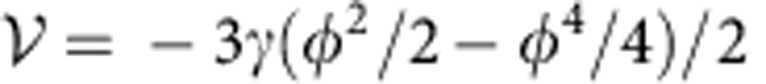. Minimization of the free-energy functional leads to explicit expressions of *μ* and *P*_*αβ*_ in terms of *ρ* and *φ*. These can then be linked to the equilibrium distribution functions 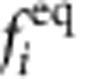 and 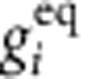. For a standard discussion of the lattice-Boltzmann algorithm the reader is referred to ref. 39.

Simulations were carried out in a rectangular channel of length *L*=150 and height *H*=100 in lattice units. The top and bottom walls were implemented using standard bounce-back boundary conditions for the distribution functions. Periodic boundary conditions were imposed along the longitudinal direction. The initial configuration consisted of each of the two phases occupying half of the domain along the longitudinal direction. The material parameters, in lattice units, were fixed to *ρ*=1, *M*=1.0, *γ*=0.005, *ξ*=1 and 
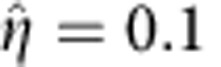
. To drive the deformation of the interface, a shear rate between the two plates *U*/*H*=2 × 10^−5^ was imposed. The simulations were run for 5 × 10^5^ steps, after which a well-developed steady state was obtained.

## Additional information

**How to cite this article:** Setu, S. A. *et al.* Superconfinement tailors fluid flow at microscales. *Nat. Commun.* 6:7297 doi: 10.1038/ncomms8297 (2015).

## Supplementary Material

Supplementary Movie 1 Front destabilisation in superconfinement.

Supplementary Movie 2 Periodic drop emission in superconfinement.

Supplementary Movie 3 Concurrent drop emission.

## Figures and Tables

**Table 1:** Data presented in Fig. 1b.

***H*** (***μ*****m)**	***N***	U^*^ **(μm s**^**−1**^**)**	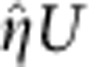 ^*^/γ
17	1	4.43	1.62
14	3	5.01±0.30	1.84±0.22
10	12	5.96±0.57	2.19±0.42
8	10	6.75±0.29	2.48±0.11

In this table, *H* is the thickness of the channel, *N* is the number of measurements, U* is the critical speed, 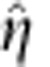 is the average viscosity of the fluids and *γ* is the interfacial tension.

**Table 2:** Data presented in Fig. 4d.

***H***** (μm)**	***N***	R^*^ **(μm)**	κ^*^ **(μm**^**−1**^**)**
17	1	4.08	0.24
14	3	3.71±0.47	0.27±0.03
10	12	2.63±0.96	0.38±0.14
8	10	2.36±0.28	0.42±0.05

In this table, *H* is the thickness of the channel, *N* is the number of measurements, R* is the radius of curvature at the critical point measured at the tip of the front and κ*=1/R* is the interface curvature.

**Table 3:** Data presented in Fig. 5b,c.

***H*** **(μm)**	***N***	***U*** **(μm s**^**−1**^**)**	***V*** **(μm s**^**−1**^**)**	***r***_**||**_ **(μm)**	***h***_**f**_ **(μm)**	***t***_**s**_ **(s)**
14	3	5.84±0.42	3.84±0.16	7.3±1.48	7.20±0.27	18.43±4.17
10	12	6.55±1.55	4.96±1.11	5.9±0.73	6.11±0.41	7.57±1.30
8	10	7.51±1.23	4.61±0.26	5.2±0.75	5.64±0.14	5.21±2.03

In this table, *H* is the thickness of the channel, *N* is the number of measurements, *U* is the speed of the leading front, *V* is the speed of the contact line, *r*_||_ is the radius of curvature in the *xy* plane, measured at the tip of the front, *h*_f_ is the thickness of the film and *t*_s_ is the average snap-off time of a detaching droplet.
